# From Dinosaurs to Modern Bird Diversity: Extending the Time Scale of Adaptive Radiation

**DOI:** 10.1371/journal.pbio.1001854

**Published:** 2014-05-06

**Authors:** Daniel Moen, Hélène Morlon

**Affiliations:** Institut de Biologie (IBENS), École Normale Supérieure, Paris, France

## Abstract

What explains why some groups of organisms, like birds, are so species rich? And what explains their extraordinary ecological diversity, ranging from large, flightless birds to small migratory species that fly thousand of kilometers every year? These and similar questions have spurred great interest in adaptive radiation, the diversification of ecological traits in a rapidly speciating group of organisms. Although the initial formulation of modern concepts of adaptive radiation arose from consideration of the fossil record, rigorous attempts to identify adaptive radiation in the fossil record are still uncommon. Moreover, most studies of adaptive radiation concern groups that are less than 50 million years old. Thus, it is unclear how important adaptive radiation is over temporal scales that span much larger portions of the history of life. In this issue, Benson et al. test the idea of a “deep-time” adaptive radiation in dinosaurs, compiling and using one of the most comprehensive phylogenetic and body-size datasets for fossils. Using recent phylogenetic statistical methods, they find that in most clades of dinosaurs there is a strong signal of an “early burst” in body-size evolution, a predicted pattern of adaptive radiation in which rapid trait evolution happens early in a group's history and then slows down. They also find that body-size evolution did not slow down in the lineage leading to birds, hinting at why birds survived to the present day and diversified. This paper represents one of the most convincing attempts at understanding deep-time adaptive radiations.

*“It is strikingly noticeable from the fossil record and from its results in the world around us that some time after a rather distinctive new adaptive type has developed it often becomes highly diversified.”* – G. G. Simpson ([1], pp. 222–223)

George Gaylord Simpson was the father of modern concepts of adaptive radiation—the diversification of ecological traits in a rapidly speciating group of organisms (Figure 1; [2]). He considered adaptive radiation to be the source of much of the diversity of living organisms on planet earth, in terms of species number, ecology, and body form [1]–[3]. Yet more than 60 years after Simpson's seminal work, the exact role of adaptive radiation in generating life's extraordinary diversity is still an open and fundamental question in evolutionary biology [3],[4].

**Figure 1:**
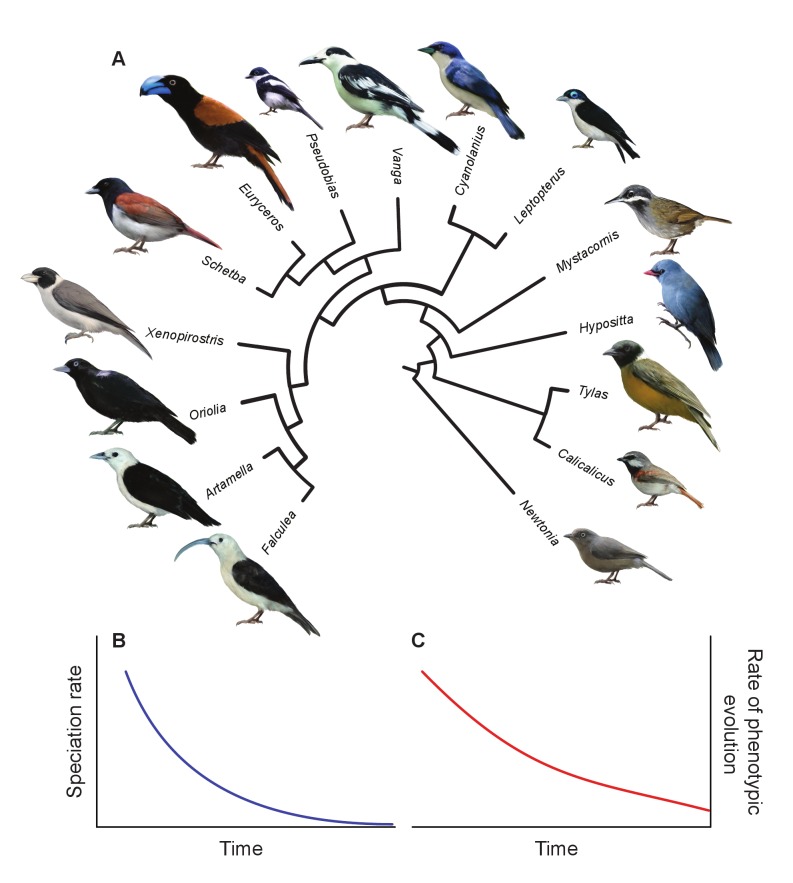
An example of adaptive radiation and early bursts in rates of speciation and phenotypic evolution. (a) The adaptive radiation of the modern bird clade Vanginae, which shows early rapid speciation, morphological diversity, and diversity in foraging behavior and diet [15],[32]. (b) Hypothetical curve of speciation rates through time that would be expected in adaptive radiation. The exponential decline in speciation rates shows that there was an “early burst” of speciation at the beginning of the clade's history. (c) Hypothetical curve of rates of phenotypic evolution through time that would be expected in adaptive radiation, also showing an early burst of evolution with high initial rates. Part (a) is reproduced from [32] with permission (under CC-BY) from the Royal Society and the original authors.

To address this question, researchers have looked for signatures of past adaptive radiation in the patterns of diversity in nature. In particular, it has been suggested that groups that have undergone adaptive radiation should show an “early-burst” signal in both rates of lineage diversification and phenotypic evolution through time—a pattern in which rates of speciation and phenotypic evolution are fast early in the history of groups and then decelerate over time (Figure 1; [3]–[5]). These predictions arise from the idea that clades should multiply and diversify rapidly in species number, ecology, and phenotype in an adaptive radiation and that rates of this diversification should decrease later as niches are successively occupied [2].

Early bursts have been sought in both fossils and phylogenies. Few fossil studies have discussed their results in the context of adaptive radiation (but see [6]), but they often have found rapid rises in both taxonomic and morphological diversity early in the history of various groups [7], ranging from marine invertebrates [8] to terrestrial mammals [9]. However, fossils often lack the phylogeny needed to model how evolution has proceeded [7]. On the other hand, studies that test for early bursts in currently existing (extant) species typically use phylogenies, which allow us to model past evolution in groups with few or no fossils [5]. Phylogenies have most often been used to test early bursts in speciation (see, e.g., [10]). However, such tests may be misled by past extinction, which will decay the statistical signal of rapid, early diversification [11]. Furthermore, diverse evolutionary scenarios beyond adaptive radiation can give rise to early bursts in speciation [12]. By contrast, studies of phenotypic diversification may be more robust to extinction [13] and they test the distinguishing feature that separates adaptive from nonadaptive radiation [2],[12].

Thus, studies of adaptive radiation in extant organisms increasingly have focused on phylogenetic tests of the early-burst model of phenotypic evolution. Some studies show strong support for this prediction in both birds [14],[15] and lizards [5],[16]. However, the most extensive study to date showed almost no support for the early-burst model. In this study, Harmon et al. [17] examined body size in 49 (and shape in 39) diverse groups of animals, including invertebrates, fishes, amphibians, reptiles, birds, and mammals. They found strong support for the early-burst model in only two of these 88 total datasets.

This result raises an important question: if adaptive radiation explains most of life's diversity [1], how is it possible that there is so little phylogenetic evidence for early bursts of phenotypic evolution? One possibility is that early bursts are hard to detect. This can be due to low statistical power in the most commonly employed tests [18]. It may also be due to a lack of precision in the way “early burst” is defined (and thus tested), as the ecological theory of adaptive radiation suggests that the rate of phenotypic evolution will decrease as species diversity increases in a group, not just over time [14],[16]. Indeed, recent studies [14],[16] detected a decline in rates with species diversity in clades that were also in the Harmon et al. [17] study, yet for which no decline over time was detected.

A second possible reason for why early-burst patterns are uncommon is more fundamental: the patterns of phenotypic diversity that result from adaptive radiation may be different at large time scales. Many of the best examples of adaptive radiation are in groups that are relatively young, including Darwin's finches (2.3 million years old [myr]; [19]) and Lake Malawi and Victoria cichlids (2.3 myr; [20]), whereas most groups that are examined for early bursts in phenotypic evolution are much older (e.g., 47 of 49 in Harmon et al. [17]; mean ± sd = 23.8±29.2 myr). So there may be an inherent difference between what unfolds over the relatively short time scales emphasized by Schluter [2] and what one sees at macroevolutionary time scales (see [21] for an in-depth discussion of this idea as it relates to speciation).

The time scale over which adaptive radiations unfold has been little explored. As a result, the link between extant diversity and major extinct radiations remains unclear. Simpson [1] believed that adaptive radiation played out at the population level, but that it should manifest itself at larger scales as well—up to phyla (e.g., chordates, arthropods). He suggested that we should see signals of adaptive radiations in large, old clades because they are effectively small-scale adaptive radiation writ large [1]. Under this view, we should see the signal of adaptive radiation even in groups that diversified over vast time scales, particularly if adaptive radiation is as important for explaining life's diversity as Simpson [1] thought it was.

Part of the reason why potential adaptive radiations at deep time scales remain poorly understood is that studies either focus on fossils or phylogenies, but rarely both. In this issue, Benson et al. [22] combine these two types of data to address whether dinosaurs show signs that they adaptively radiated. Unlike most other studies, the temporal scale of the current study is very large—in this case, over 170 million years throughout the Mesozoic era, starting at 240 million years ago in the Triassic period. This characteristic allowed Benson et al. to shed light on deep-time adaptive radiation.

The authors estimated body mass from fossils by using measurements of the circumference of the stylopodium shaft (the largest bone of the arm or leg, such as the femur), which shows a consistent scaling relationship with body mass in extant reptiles and mammals [23]. They then combined published phylogenies to obtain a composite phylogeny for the species in their body-size dataset. The authors finally conducted two types of tests of the rate of body-size evolution—tests of early bursts in phenotypic evolution that are the same as those of Harmon et al. [17], as well as an additional less commonly used test that estimates whether differences between estimated body size at adjacent phylogenetic nodes decreases over time.

Benson et al. [22] found two striking results. First, in both of their analyses, the early-burst model was strongly supported for most clades of dinosaurs. This early burst began in the Triassic period, indicating that diversification in body size in dinosaurs began before the Triassic-Jurassic mass extinction event would have opened competition-free ecological space (as commonly hypothesized; [24],[25]). Rather, the authors [22] suggest that a key innovation led to this rise in dinosaurs, though it is not clear what this innovation was [26]. In general, though, the finding of an early burst in body-size evolution in most dinosaurs—if a consequence of adaptive evolution—suggests that adaptive radiation may play out over large evolutionary time scales, not just on the short time scales typical of the most well-studied cases of extant groups.

Second, one clade—Maniraptora, which is the clade in which modern-day birds are nested—was the only part of the dinosaur phylogeny that did not show such a strong early burst in body-size evolution. Instead, this clade fit a model to a single adaptive peak—an optimum body size, if you will—but also maintained high rates of undirected body-size evolution throughout their history. Benson et al. [22] suggest that this last result connects deep-time adaptive radiation in the dinosaurs, which quickly exhausted the possibility of phenotypic space, with the current radiation in extant birds, which survived to the present day because their constant, high rate of evolution meant that they were constantly undergoing ecological innovation. This gives a glimpse into why modern birds have so many species (an order of magnitude higher than the nonavian dinosaurs) and so much ecological diversity.

The use of fossils allowed Benson et al. [22] to address deep-time radiation in dinosaurs and its consequence on present-day bird diversity. Nevertheless, the promise of using fossils to understand adaptive radiation has its limits. The paleontological dataset presented here is exceptional, yet still insufficient to explore major components of adaptive radiations like actual ecological diversification. As in many paleontological studies, Benson et al. used body-size data to represent ecology because body size is one of the few variables that is available for most species. But it is unclear how important body size really is for ecological diversification and niche filling, because body size is important for nearly every aspect of organismal function. Consequently, evolutionary change in body size can result not only from the competition that drives adaptive radiation, but also from predation pressure, reproductive character displacement, and physiological advantages of particular body sizes in a given environment, among other reasons [27].

Despite the broad coverage of extinct species presented in Benson et al. [22], the data were insufficient to study another major part of adaptive radiation: early bursts of lineage diversification. While new approaches are becoming available to study diversification with phylogenies containing extinct species [28],[29] or with incomplete fossil data [30], these approaches are limited when many taxa are known from only single occurrences. This is the case in the Benson et al. dataset, and more generally in most fossil datasets.

Given that few fossils exist for many extant groups, a major goal for future studies will be the incorporation of incomplete fossil information into analyses primarily focused on traits and clades for which mostly neontological data are available. For example, Slater et al. [31] developed an approach to include fossil information in analyses of phenotypic evolution. They showed that adding just a few fossils (12 fossils in a study of a 135-species clade) drastically increased the power and accuracy of their analyses of extant taxa. Thus, the combination of fossil data and those based on currently living species is important for future studies, as are new approaches that allow analyzing early bursts of lineage diversification along with phenotypic evolution in fossils.

So what answers do Benson et al. [22] bring to Simpson's original question of the importance of adaptive radiation for explaining diversity on earth? The authors present an intriguing and unconventional link between adaptive radiation and the diversity of modern-day birds. They argue that bird diversification was possible because the dinosaur lineage leading to birds did not exhaust niche space, potentially thanks to small body sizes; in contrast, other dinosaur groups adaptively radiated, filled niche space, and thus could not produce the ecological innovation that may have been necessary to survive the Cretaceous-Paleogene mass extinction. This intriguing hypothesis suggests an important role for the relative starting points of successive adaptive radiations in explaining current diversity, giving a new spin to the pivotal question raised by Simpson more than 60 years ago.

## Abbreviations

myr: million years old
